# The Simplest Integrated Multicellular Organism Unveiled

**DOI:** 10.1371/journal.pone.0081641

**Published:** 2013-12-11

**Authors:** Yoko Arakaki, Hiroko Kawai-Toyooka, Yuki Hamamura, Tetsuya Higashiyama, Akira Noga, Masafumi Hirono, Bradley J. S. C. Olson, Hisayoshi Nozaki

**Affiliations:** 1 Department of Biological Sciences, Graduate School of Science, University of Tokyo, Bunkyo-ku, Tokyo, Japan; 2 Division of Biological Science, Graduate School of Science, Nagoya University, Nagoya, Aichi, Japan; 3 Institute of Transformative Bio-Molecules (WPI-ITbM), Nagoya University, Nagoya, Aichi, Japan; 4 JST ERATO Higashiyama Live-Holonics Project, Nagoya University, Nagoya, Aichi, Japan; 5 Division of Biology, Kansas State University, Chalmers, Manhattan, Kansas, United States of America; University of Bern, Switzerland

## Abstract

Volvocine green algae represent the “evolutionary time machine” model lineage for studying multicellularity, because they encompass the whole range of evolutionary transition of multicellularity from unicellular *Chlamydomonas* to >500-celled *Volvox*. Multicellular volvocalean species including *Gonium pectorale* and *Volvox carteri* generally have several common morphological features to survive as integrated multicellular organisms such as “rotational asymmetry of cells” so that the cells become components of the individual and “cytoplasmic bridges between protoplasts in developing embryos” to maintain the species-specific form of the multicellular individual before secretion of new extracellular matrix (ECM). However, these morphological features have not been studied in the four-celled colonial volvocine species *Tetrabaena socialis* that is positioned in the most basal lineage within the colonial or multicellular volvocine greens. Here we established synchronous cultures of *T. socialis* and carried out immunofluorescence microscopic and ultrastructural observations to elucidate these two morphological attributes. Based on immunofluorescence microscopy, four cells of the mature *T. socialis* colony were identical in morphology but had rotational asymmetry in arrangement of microtubular rootlets and separation of basal bodies like *G. pectorale* and *V. carteri*. Ultrastructural observations clearly confirmed the presence of cytoplasmic bridges between protoplasts in developing embryos of *T. socialis* even after the formation of new flagella in each daughter protoplast within the parental ECM. Therefore, these two morphological attributes might have evolved in the common four-celled ancestor of the colonial volvocine algae and contributed to the further increase in cell number and complexity of the multicellular individuals of this model lineage. *T. socialis* is one of the simplest integrated multicellular organisms in which four identical cells constitute the individual.

## Introduction

Organisms on Earth exhibit a wide array of morphological and genetic diversity. This diversity originates from evolution of organisms since the origin of life on the Earth, via evolutionary transitions in individuality (ETIs), in which individuals gathered to become different individuals of higher-level [1]. According to Michod [1], the major landmarks of diversification and hierarchical organization of organisms passed through serial steps of ETIs: from genes to first cells, from prokaryotic cells to eukaryotic cells, from independent unicellular cells to controlled multicellular organisms, from asexual to sexual populations, from solitary to social organisms.

**Figure 1:**
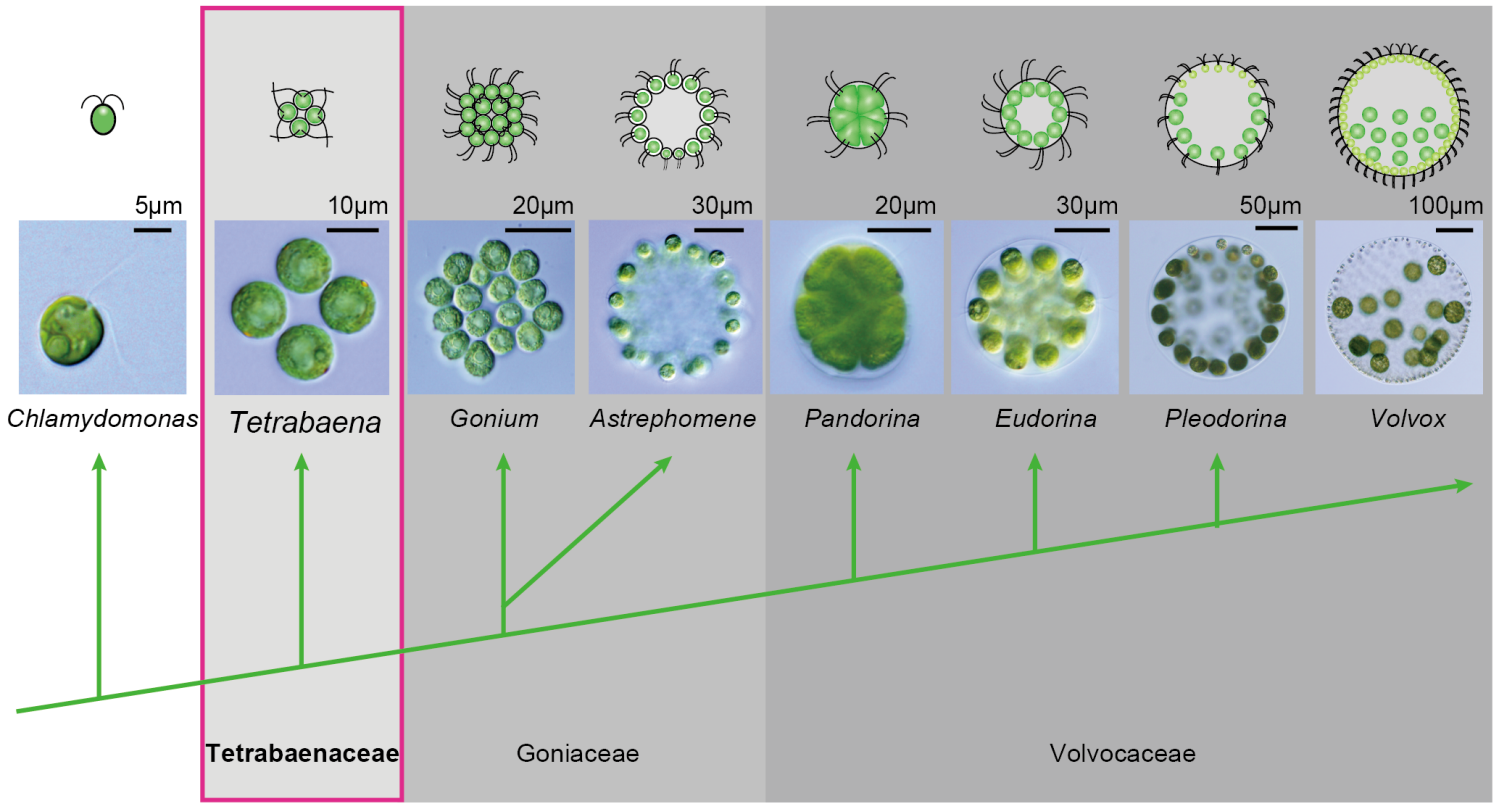
Rough outline of phylogenetic relationships in volvocine green algae [9], [10], [15].

Multicellularity is an evolutionary transition that has occurred more than twenty-five times in distinct eukaryotic lineages [2], [3]. However, in most lineages, the evolutionary signature of the transition to multicellularity is obscured because most lineages lack related species that have maintained the ancestral transitional forms from unicellular to multicellular possibly due to extinction or lack of discovery. Alternatively, the volvocine green algae include a complete range of ETIs, from unicellular *Chlamydomonas* to multicellular *Volvox* [4]–[6]. Furthermore, the genomes of *Chlamydomonas reinhardtii* [7] and *Volvox carteri* [8] have been sequenced, and phylogeny within this group is well resolved [9], [10], and culture and molecular genetic methods have been established [11]–[13]. Thus the volvocine algae offer an excellent opportunity for studying multicellular evolution (Figure 1).

Colonial volvocine algae or colonial Volvocales constitute a robust monophyletic group composed of three families, the Tetrabaenaceae, Goniaceae, and Volvocaceae, and Tetrabaenaceae is the most basal group suggested by morphological and molecular data [9], [10], [14], [15] (Figure 1). The Tetrabaenaceae was established by Nozaki and Ito [14] and includes two four-celled species *Tetrabaena socialis* and *Basichlamys sacculifera* [10], [16]. *T. socialis* has four *Chlamydomonas*-like cells arranged like a four-leaf clover (Figure 1) and swims with its flagellar bases orienting forwardly as the colony rotates (Video S1). The four cells constitute a square vegetative colony by the connections of their extracellular matrices (ECM) [17], [18].

The transition to multicellularity in the volvocine algae occurred about 200 million years ago in the *Chlamydomonas*-like ancestor [10]. Kirk [4] proposed that there are twelve steps for multicellularity leading to *Volvox* where six of the steps [incomplete cytokinesis, partial inversion of embryo, rotation of basal bodies (BB), establishment of organismic polarity, transformation of cell walls into an ECM, and genetic modulation of cell number] are required for the divergence of the 16-celled *Gonium pectorale* in the colonial volvocine algae. However, Kirk’s twelve-step model did not discuss the four-celled Tetrabaenaceae. Subsequently, Herron et al. [10] deduced the character evolution of the twelve steps based on the phylogenetic relationships of the volvocine algae including the Tetrabaenaceae. According to them, the unicellular ancestor at first embedded their cells in a common ECM and obtained a genetic control of cell number to become a common ancestor of three families of the colonial and multicellular volvocine algae (Volvocaceae, Goniaceae and Tetrabaenaceae). After divergence of the Tetrabaenaceae, incomplete cytokinesis, rotational asymmetry of cells (rotation of BB), and organismal polarity might have evolved in the common ancestor of the Volvocaceae and Goniaceae [10]. In the multicellular members of the volvocine algae, *G. pectorale*, *Pandorina morum* and *V. carteri*, newly formed embryos have species-specific shape due to connections or cytoplasmic bridges between the protoplasts of the developing embryos before secretion of a new ECM [19]–[21]. Furthermore, because the flagellar motion of the constitutive cells of the colonial or multicellular forms is essentially different from that of the unicellular organization in the volvocine algae, rotational asymmetry of cells might have been acquired for effective swimming of the organized cells or multicellular organism [22]. Thus, character evolution as deduced by Herron et al. [10] indicates that the Tetrabaenaceae does not have these multicellular traits and may not be considered as integrated multicellular organisms. However, there have been no cell biological or ultrastructural studies of the Tetrabaenaceae except for transmission electron microscopic (TEM) observation of *T. socialis* and *Basichlamys sacculifera* in vegetative phase [16], [18].

The present study was undertaken to evaluate the multicellular morphological traits of the most primitive colonial volvocine green *Tetrabaena socialis*, with particular regard to the cytoplasmic bridges between embryonic protoplasts and rotational asymmetry of the vegetative cells.

## Results

### Synchronous Culture of *T. socialis*

**Figure 2:**
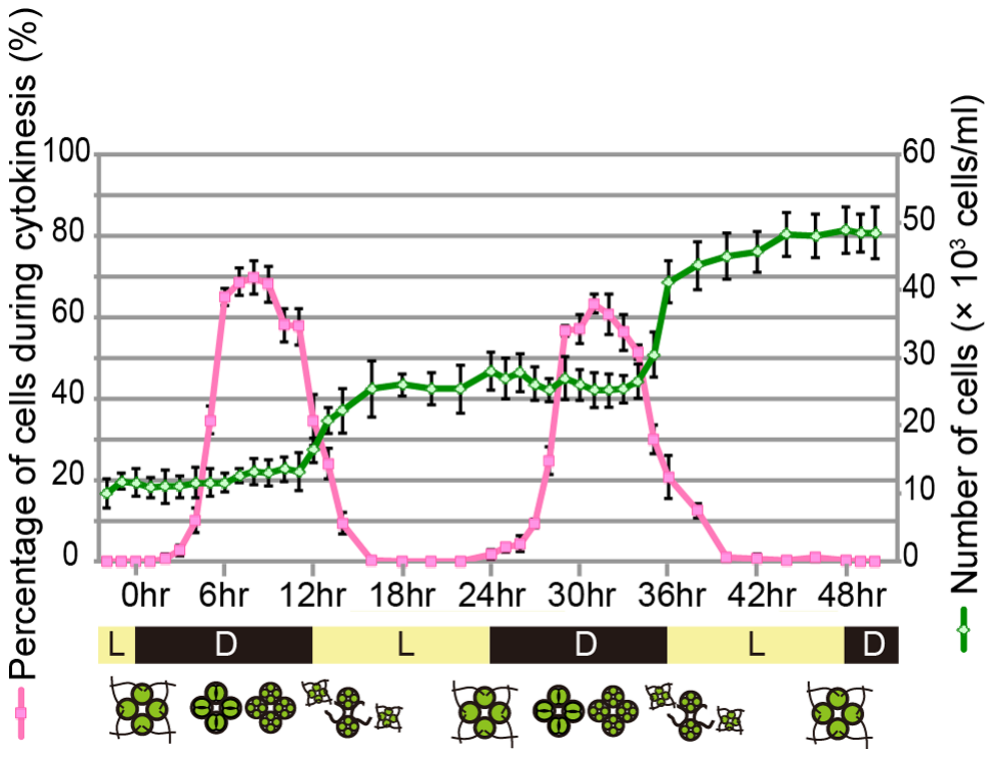
Time course of synchronous culture of *Tetrabaena socialis* NIES-571. Light-dark cycle (light:dark = 12 h:12 h) were indicated on the horizontal axis, percentages of cells during cytokinesis were indicated on a vertical line of left side with a pink line, and number of cells were indicated on a right side with a green line. Each error bars shows standard deviation (n = 3).

The cell cycle of *C. reinhardtii* through an extended G1 phase correlates with the availability of light and nutrients [13]. Having grown many times their original cell size, they must divide multiple times (S/M phase), a process known as multiple fission. Entry into multiple fission occurs during darkness, thus the *C. reinhardtii* cell cycle can be highly synchronized to light-dark cycles [13]. The life cycles of the Volvocaceae, particularly *V. carteri* can also be synchronized to light-dark cycles even though its life cycle is 48 h compared to a 24 h cycle for *C. reinhardtii* [11]. To determine if *T. socialis* can be synchronized to light-dark cycles, cultures of *T. socialis* were grown in a 12 h light-12 h dark cycle for 50 h in photoautotrophic standard *Volvox* media (SVM), where colonies are unable to grow in the dark. At hourly time points a fraction of the cells were removed, their colony concentration was calculated and the cells were visualized by microscopy to determine if they were in G1 or S/M phase (Figure 2). During the light period, nearly all observed cells were flagellated indicative that they were in G1 phase, which was confirmed by the fact that the number of colonies did not change during this time (Figure 2). By 5–6 h of the dark period, approximately 70% of cells were de-flagellated and undergoing either a first or second round of multiple fission (Figure 2). When the next light period began, small daughter colonies just hatching from the parental ECM were abundant and swimming in the culture. The mother colony remained intact during multiple fission, and that daughter colonies were formed within the mother cells. All cells in a *T. socialis* colony are capable of reproduction [17].

### *T. socialis* Microtubular Rootlets Exhibit Rotational Asymmetry

**Figure 3:**
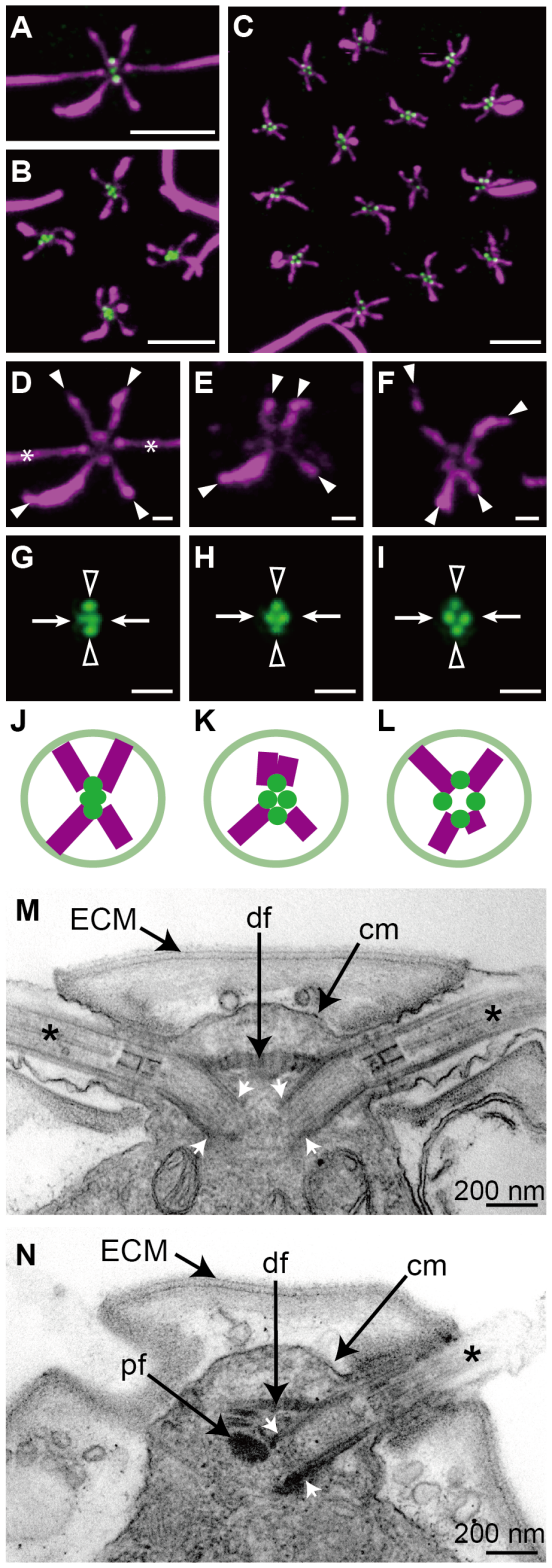
Images and diagrams of microtubular rootlet (MTR) and basal bodies (BB)/pro-basal bodies (pBB). (A–I) Immunofluorescence microscopy. (A–C) Double stained fluorescence of acetylated tubulin and CrSAS-6 showing MTR and BB/pBB, respectively. Each scale bar represents 5 µm. (A) *Chlamydomonas reinhardtii*. (B) *Tetrabaena socialis*. (C) *Gonium pectorale*. (D–F) Fluorescence of acetylated tubulin. Each white arrowhead or asterisk indicates distal end of MTR or flagellum, respectively. Each scale bar represents 1 µm. Upper sides of panels E and F represent the directions of center in the flattened colonies. (D) *C. reinhardtii*. (E) *T. socialis.* (F) *G. pectorale*. (G–I) Fluorescence of CrSAS-6. Each arrow or arrowhead indicates BB or pBB, respectively. Each scale bar represents 1 µm. Upper sides of panels H and I represent the directions of center in the flattened colonies. (G) *C. reinhardtii.* (H) *T. socialis.* (I) *G. pectorale.* (J–L) Diagrams of MTR and BB/pBB arrangements. Upper sides of panels K and L represent the directions of center in the flattened colonies. (J) *C. reinhardtii.* (K) *T. socialis.* (L) *G. pectorale.* (M, N) Transmission electron microscopy of *T. socialis*. ECM, extracellular matrix; cm, cell membrane; df, distal fiber; pf, proximal fiber; asterisk, flagellar proper. (M) Longitudinal section of anterior end of cell showing BB and distal fiber. Note proximal ends of the two BB (white arrows) are separated from each other. (N) Longitudinal section of anterior end of cell showing BB with proximal fiber.

*C. reinhardtii* cells swim with via a “breast stroke” while all other multicellular volvocine species have rotated BB and have swimming strokes that depend on the position of the cell within the colony [22]. To understand the structural evolution of colonial multicellularity in *T. socialis* compared to unicellular *C. reinhardtii* and 16-celled *Gonium pectorale*, comparative immunofluorescence microscopy of microtubular rootlets (MTR), BB and pro-basal bodies (pBB) in the three species was performed (Figure 3A–L).

In *C. reinhardtii,* cells have an X-shaped MTR exhibiting rotational symmetry and show a 60° angle between two extending MTR in *anti* and *syn* sides (Figure 3D) (figure 14 [23]). In the *T socialis* colony, the four cells were indistinguishable from one another but each cell exhibited rotational asymmetry (Figure 3E). Two MTR extending toward the center of the *T. socialis* colony was arranged nearly in parallel, whereas the other two MTR extended toward the periphery and showed a 90° angle between them. In *G. pectorale*, the peripheral cells of the 16-celled colony also exhibited rotational asymmetry, but MTR extending toward the center of the colony showed a wider angle than those extending toward the periphery of the colony (Figure 3F), whereas the central four cells of *G. pectorale* exhibited rotational symmetry of MTR similar to *C. reinhardtii* cells [24].

CrSAS-6 is a protein localized at central part of cartwheel of BB and also attached to pBB [25]. Thus, four dots immunostained with anti-CrSAS-6 antibody in each of the volvocine vegetative cells represented a pair of BB and a pair of pBB. In all of the three volvocine species examined here, two dots from which two flagella extended were closer to each other than the other two dots (Figure S1). Thus, the closer dot pair can be considered BB, whereas the other pair pBB. Based on our indirect immunofluorescence microscopic observations, pairs of BB of *C reinhardtii* were very close to each other to appear to be almost one dumbbell-shaped dot (Figure 3G) and their distance was 280±40 nm (n = 20). In contrast, two dots representing a pair of BB in *T. socialis* cells and peripheral cells of the 16-celled *G. pectorale* colonies were apparently separated from each other (Figure 3H, I). Distances between pairs of BB in the *T. socialis* and *G. pectorale* cells were 360±40 nm (n = 20) and 470±50 nm (n = 20), respectively. Distances between pairs of pBB in the three species fell within a small range (Figure S1A).

To further examine how BB are placed within *T. socialis* colonies relative to their flagella, we observed them with TEM. The two BB in the mature cell were inserted in the anterior region of the protoplast with an about 120° angle to each other and their proximal ends were separated from each other, showing a striated distal fiber and an electron-dense proximal fiber (Figure 3M, N). In *C. reihhardtii*, two BB are almost attached to each other at the proximal ends and show a 90° angle between them (figure 13 [23]).

### Cytoplasmic Bridges between Protoplasts in Developing Embryo of *T. socialis*

Cytoplasmic bridges in the volvocine algae are thought to be important for multicellularity, though it is not certain if they are structural, or for communication [21]. The simplest hypothesis is that cytoplasmic bridges between daughter protoplasts form during the multiple fission cell cycle of mother cells to form four-celled square daughter colonies. To determine if and when cytoplasmic bridges form in *T. socialis* daughter colonies, cultures were synchronized to a light-dark cycle and visualized by light microscopy and TEM as they progressed through mitosis. Also, multiple fission has been hypothesized to be modified such that incomplete cytokinesis keeps daughter cells attached as a colony. To our knowledge, this hypothesis has not been directly observed in the most basal colonial multicellular *T. socialis* relative to unicellular *C. reinhardtii*.

**Figure 4:**
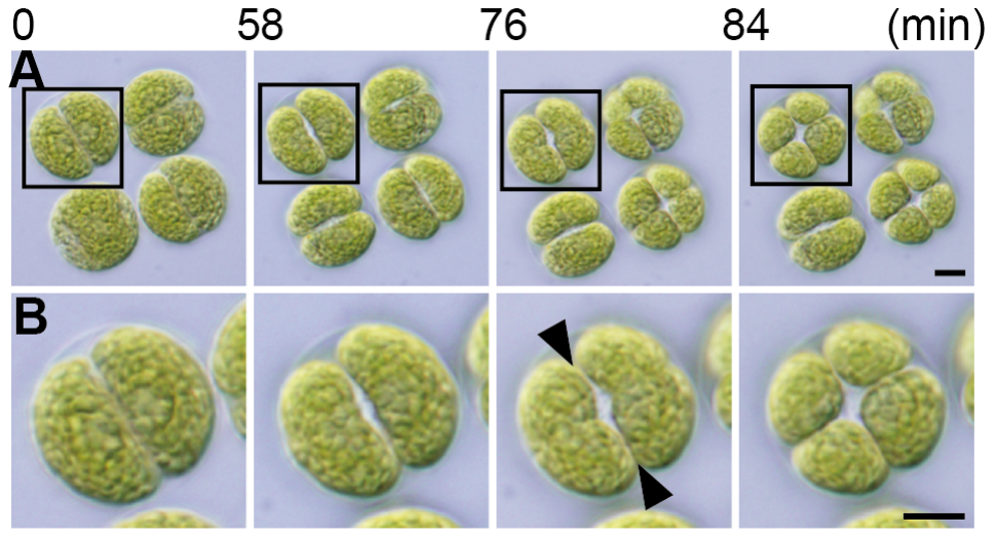
Time-lapse analysis for cytokinesis of *Tetrabaena socialis* NIES-571. (A) Parental colony of *T*. *socialis*, shown at the same magnification throughout. Scale bar represents 5 µm. (B) Enlarged image in frame in (A), shown at the same magnification throughout. Scale bar represents 5 µm. Note possible connections between daughter protoplasts (arrowheads).

In the initial stage of daughter colony formation of *T. socialis*, each parental cell lost its flagella by shortening or resorption of them. The cell then divided into four daughter protoplasts by two successive longitudinal divisions (Figure 4, 0–58 min). The second division was perpendicular to the first to form four daughter protoplasts arranged like a four-leaf clover (Figure 4, 76–84 min). Each daughter protoplast then grew two new flagella within the parental ECM. During these processes, the four daughter protoplasts maintain the four-leaf clover-like form without significant movement from one another based on our time-lapse analysis (Figure 4). This result suggests that *T. socialis* daughter protoplasts are possibly connected via cytoplasmic bridges until the daughter colony matures by the formation of new ECM.

**Figure 5:**
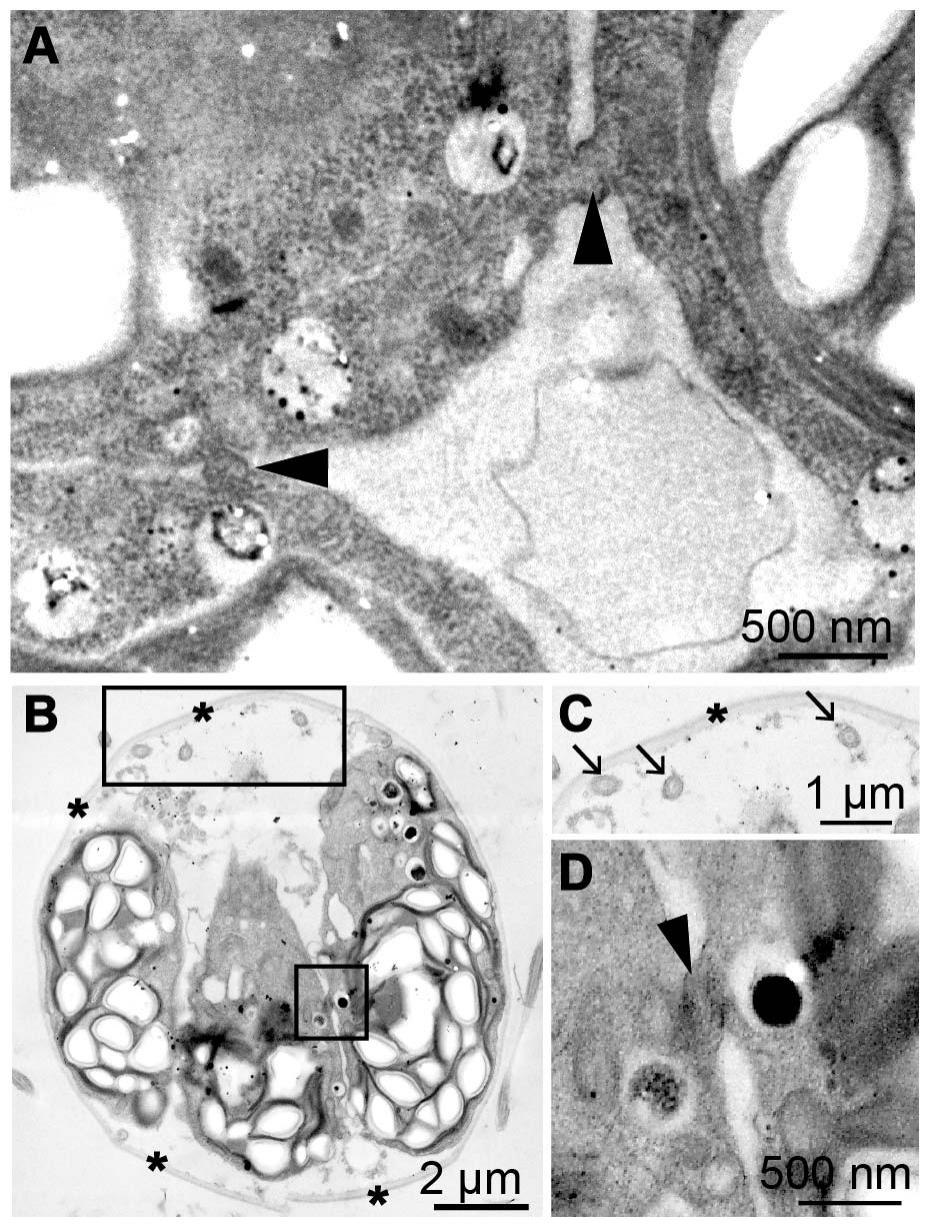
Transmission electron microscopy of cytokinesis of four-celled embryos of *Tetrabaena socialis* NIES-571. (A) Transverse section of almost central part of four daughter protoplasts before formation of new flagella. Note a daughter protoplast connected to two neighbors by cytoplasmic bridges (arrowheads). (B) Semi-longitudinal section of daughter protoplasts after formation of new flagella (large frame) within parental extracellular matrix (ECM) (asterisks). Note two protoplast connected to each other by cytoplasmic bridges (small frame). (C, D) Enlarged images of two frames in (B). (C) New flagella (arrows) within parental ECM (asterisks). (D) Cytoplasmic bridges (arrowhead) connecting two daughter protoplasts.

To determine if cytoplasmic bridges were present in daughter *T. socialis* colonies, synchronous cultured cells entering the dark period and were 70% mitotic, were observed by TEM (Figure 5). In the four-celled embryo, cytoplasmic bridges were observed in both sides facing the adjoining protoplasts (Figure 5A, indicated by arrowheads). Because the second cleavage of *T. socialis* is perpendicular to first cleavage under the light microscope (Figure 4), one side was formed by the first cleavage whereas the other by the second cleavage. Such cytoplasmic bridges remained even after formation of new flagella within the parental ECM (Figure 5B–D).

These results suggest that cytoplasmic bridges may play an important role in conjunction with ECM deposition in keeping *T. socialis* daughter colonies multicellular.

### Eyespots in *T. socialis*

The asymmetric placement of the *C. reinhardtii* eyespot is essential for cells to properly swim toward light [11], and contributes to its “breast stroke” swimming movement. Presently, the placement of eyespots within colonial volvocalean species is well known [22], although the replacement of eyespots in the tetrabaenaean colonies have remained ambiguous [17]. Moreover, *C. reinhardtii* has 2–3 layers of eyespot globules [26], whereas more complex colonial volvocalean species 4–8 eyespot layers [11], [27]–[29]. We sought to investigate how eyespots are placed within colonial Volvocales, and to determine where in the clade 4–8 layered eyespots evolved.

**Figure 6:**
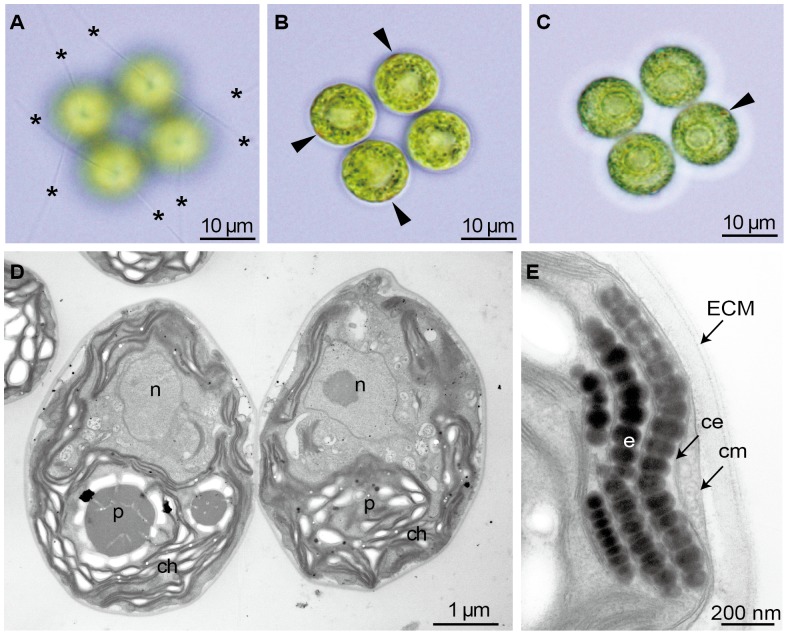
Light and transmission electron microscopy of vegetative colonies of *Tetrabaena socialis* NIES-571. (A–C) Three light microscopic views of four-celled vegetative colony, showing positions of flagella and eyespots. Note that flagella (asterisks) and eyespots (arrowheads) are arranged in symmetric pattern in the whole colony. (D, E) Transmission electron microscopy. ECM, extracellular matrix; ce, chloroplast envelope; ch, chloroplast; cm, cell membrane; n, nucleus; p, pyrenoid. (D) Longitudinal section of vegetative colony. (E) Eyespot composed three layers of globules.

Under light microscope, all four cells of *T. socialis* vegetative had identical placements in flagella and eyespots (Figure 6A–C). The four eyespots were arranged with rotational symmetry in the whole four-celled colony, but show rotational asymmetry in each cell because each eyespot was always positioned in the *cis-syn* side of each cell as in other colonial or multicellular volvocine algae [22].

Using TEM on vegetative cells, *T. socialis* cellular structure was very similar to that of a vegetative *C. reinhardtii* cell [13]; with a cup-shaped chloroplast occupied the peripheral region of the protoplast and a nucleus was centrally located (Figure 6D). The chloroplast contained a large pyrenoid in the bottom and an eyespot in the anterior periphery, again similar to *C. reinhardtii* cells and supporting the close evolutionary relation of the two species [9], [10], [15]. In *T. socialis*, the eyespot was concave, and composed of two or three layers of electron-dense globules lying just beneath the chloroplast membrane (Figure 6E). The innermost of the three layers was often discontinuous in section.

## Discussion

### Asymmetrical Cells and Cytoplasmic Bridges between Daughter Protoplasts in the Volvocine Algae

The present immunofluorescence microscopic observations clearly demonstrated that the four-celled colony of *T. socialis* had cells with rotational asymmetry and separated BB (Figures 3). These two situations are essentially the same as those of other multicellular volvocalean species of the Goniaceae and Volvocaceae, *G. pectorale* (figures 31–33 [24]), *Astrephomene gubernaculifera* [30], *Platydorina caudata* [31], and *V. carteri* [32]. In contrast, cells of *C. reinhardtii* exhibit rotational symmetry in arrangement of MTR and have adjacent BB (Figure 3) as previously described by Ringo (figures 13 and14 [23]) and Preble et al. [33]. Previous studies demonstrated that peripheral cells of *G. pectorale* and cells of *V. carteri* beat two flagella in nearly the same direction so that they can swim effectively as cooperative multicellular organisms, whereas in the unicellular species *C. reinhardtii* cells beat their flagella like breast stroke so that the unicells can swim effectively [34], [35]. Rotational asymmetry of MTR in cells might be important for multicellularity in volvocine algae [4] and arrangements of MTR are related to flagellar beating pattern [35]. Thus, the asymmetrical arrangement of MTR and separated BB in *T. socialis* may indicate that each cell has a role for colonial motility as the component of the multicellular individual. However, the mode of MTR asymmetry in the *T. socialis* colony is different from that of other colonial or multicellular volvocine algae (Goniaceae and Volvocaceae). In peripheral cells of the flattened 16-celled colony of *G. pectorale*, the angle of two MTR extending to the center of the colony is wider than that of the other two MTR extending exteriorly (Figure 3F). MTR arrangements of mature cells of complex spheroidal colonies of *A. gubernaculifera* [30] and *V. carteri* [32] are essentially the same as those of *G. pectorale* in having almost parallel MTR positioned in *syn* side (exterior side of the *G. pectorale* flattened colony) and their MTR are significantly asymmetrical. In contrast, two MTR extending to the center of *T. socialis* colony were nearly parallel whereas the other two MTR extending to the colonial periphery were arranged with a 90° angle to each other (Figure 3E). In addition, two BB in mature vegetative cells of *T. socialis* are different from those of other colonial volvocalean members. The two BB of *T. socialis* are arranged with a 120° angle to each other (Figure 3M) and bear an electron-dense proximal fiber (Figure 3N). In contrast, those of *G. pectorale* [24], *A. gubernaculifera* [30], *P. caudata* [31] and *V. carteri* [32] are nearly parallel and lack a proximal fiber.

### Cytoplasmic Bridges are Formed during Multiple Fission of Mother Cells in *T. Socialis*

The present TEM observations clearly demonstrate the presence of cytoplasmic bridges between daughter protoplasts that form during the process of multiple fission and persist after the maturation of the colony when they form new flagella (Figure 5). Thus *T. socialis*-specific cell arrangement in a colony should be determined by means of the cytoplasmic bridges before the formation of new ECM during daughter colony formation as observed in *G. pectorale* [19], *A. gubernaculifera* [36], *Pandorina morum* [20], *Eudorina elegans* [37], *Platydorina caudata* [38], and *V. carteri* [21]. Moreover, these results suggest that the evolution of cytoplasmic bridges may be a key structural innovation required to keep colonies together and thus multicellular.

Two morphological characteristics observed in *T. socialis* (rotational asymmetry of cells with separated BB and cytoplasmic bridges between daughter protoplasts) are considered important for multicellularity in volvocine lineage, and they are common to goniacean and volvocacean species such as *G. pectorale* and *V carteri* [5], [10]. Based on the morphological and multi-gene sequence data [9], [10], [15], [16], the Tetrabaenaceae (including *T. socialis*) is basal or sister to the clade composed of other members of the colonial Volvocales (Goniaceae and Volvocaceae). Therefore, the two key characteristics, rotational asymmetry of cells with separated BB and cytoplasmic bridges during embryogenesis, might have been acquired before the divergence of the four-celled Tetrabaenaceae (Figure 1) ca. 200 MA in the common ancestors of the extant colonial or multicellular volvocine green algae [10]. However, the fact that only *T. socialis* has parallel MTR in *anti* side (see above) and its sister relationship to other colonial or multicellular volvocine algae [9], [10] might suggest independent evolutions of the MTR asymmetry in these two sister lineages.

### Origins of Multilayered Eyespots in the Volvocine Algae

Our TEM observations resolved that the vegetative cells of *T. socialis* has an eyespot which consists of two or three layers of electron-dense globules like *C. reinhardtii* [26]. In more advanced colonial or multicellular volvocalean species, *Volvulina steinii*, *V. pringsheimii*, *Platydorina caudata*, [27], *Eudorina illinoiensis* [28], *Volvox tertius* [29], and *V. carteri* [11], eyespots composed of four to eight layers of globules were observed. In these advanced multicellular species, eyespots in the anterior cells are larger than those in the posterior cells of the same colony, because of effective phototaxis [39]. As discussed above, the Tetrabaenaceae is basal to the clade composed of these advanced members of the colonial Volvocales (Figure 1). Thus, evolution of the eyespots of four or more layers of globules might have occurred in the ancestor(s) of the multicellular volvocine algae after the divergence of the ancestor of the Tetrabaenaceae. However, the eyespot of *T. socialis* has a concave anterior face as in other multicellular volvocalean species [27]–[29] unlike that of *C. reinhardtii* [26].

## Conclusions and Perspectives

The present study clearly demonstrated that *T. socialis* has rotational asymmetry of cells and cytoplasmic bridges between daughter protoplasts. Thus, *T. socialis* is one of the simplest integrated multicellular organisms in which four identical cells constitute the individual [2], [40], [41]. Because the Tetrabaenaceae is basal to other multicellular volvocine greens (Goniaceae and Volvocaceae) including the complex organism *Volvox* (Figure 1), five of the first six steps of Kirk’s twelve steps [4] (incomplete cytokinesis, rotation of BB, establishment of organismic polarity, transformation of cell walls into an ECM, and genetic modulation of cell number) might have been attained in the four-celled common ancestor of the extant multicellular volvocine greens. Further morphological and molecular analyses of *T. socialis* will provide fundamental bases that directly affected the evolution of multicellularity and complexity of the individuals in this model lineage.

## Materials and Methods

### Cultures

Strains used in this study are listed in Table 1. *Chlamydomonas reinhardtii* C-239 was maintained synchronously in 300 mL tris-acetate-phosphate medium [13] in a silicon-capped 500 mL flask with aeration at 25°C, on a 12 h light and 12 h dark schedule under cool-white fluorescent lamps at an intensity of 110–150 µmol⋅m^–2^⋅s^–1^. In order to establish synchronous cultures of *Tetrabaena socialis* NIES-571, cells were grown in 300 mL SVM [42] in a silicon-capped 500 mL flask with aeration at 20°C, on a 12 h light and 12 h dark schedule under cool-white fluorescent lamps at an intensity of 110–150 µmol⋅m^–2^⋅s^–1^. Percentages of *T. socialis* cell dividing during daughter colony formation and cell density of the culture were counted 24 h after the inoculation under the light microscope, for every one hour during dark period and every two hours during light period. Experiments were repeated three times. Daughter colonies before hatching from parental ECM were considered “one cell” in counting cell densities. *Gonium pectorale* K4-F3-4 (mating type *plus*, one of the F3 backcross strains to K41 [mating type *plus*] originating from K41×K32 [F1 strains of Kaneko3×Kaneko4] [43], [44]) was cultured in SVM as described above for *T. socialis*.

**Table 1:** List of three volvocine algae used in this study.

Vegetative phase	Straindesignation	Scientific name
Unicell	C-239 (wild type)	*Chlamydomonas reinhardtii*
4-celled colony	NIES-571	*Tetrabaena socialis*
16-celled colony	K4-F3-4	*Gonium pectorale*

### Indirect Immunofluorescence Microscopy

MTR arrangements and distances between a pair of BB were observed by immunostaining modified from Nishii et al. [12]. Mature cells of *C. reinhardtii* C-239, *T. socialis* NIES-571 and *G. pectorale* K4-F3-4 were selected from synchronous cultures, attached to polyethylenimine (PEI) coated coverslips and fixed with 3.7% formaldehyde (Sigma Aldrich, St. Louis, MO, USA), 0.1% TritonX-100 (Sigma Aldrich), 1 mM DTT (Nacalai Tesque Inc., Kyoto, Japan) in phosphate-buffered saline (PBS). After fixation, chlorophyll was extracted in extracting solution (1% IGEPAL CA-630 [Sigma Aldrich], 1% BSA [Sigma Aldrich], 1 mM DTT in PBS). The fixed cells were incubated in block buffer-A (2.2% Gelatin [Sigma Aldrich], 0.05% NaN_3_, 5% BSA [Sigma Aldrich] in PBS), and then incubated in block buffer-B (10% Goat Serum [Sigma Aldrich] in buffer-A) for blocking. The cells were then subjected to two primary antibodies (for 1 h at 37°C) and two secondary antibodies (for 1 h at 37°C). The primary antibodies were monoclonal anti-acetylated tubulin (clone 6-11B-1, Sigma Aldrich) for MTR [45] and rabbit anti-CrSAS-6 [25] for BB diluted in 1∶500 and 1∶300, respectively, with 20% block buffer-A in TPBS (0.1% Tween 20 [Sigma Aldrich] in PBS). The secondary antibodies were goat anti-mouse Alexa Fluor 568 (Invitrogen, Carlsbad, CA, USA) and goat anti-rabbit Alexa Fluor 488 (Invitrogen) that were diluted in 1∶500 with TPBS.

Confocal images were obtained with an LSM 780 (Carl Zeiss, Jena, Germany). Distances between a pair of BB and between a pair of pBB were measured using ImageJ 1.45s (National Institutes of Health, Bethesda, MD, USA). Western blotting analyses were carried out as described by Nakazawa et al. [25] for evaluation of the specificity of the anti-SAS-6 antibody (Figure S2).

### Time-lapse Microscopy

Synchronous cultured *T. socialis* NIES-571 colonies were attached to PEI-coated coverslips, put on slides and sealed with Vaseline to avoid water evaporation. Preparations were observed by a BX-60 microscope (Olympus, Tokyo, Japan) with DP Controller 1. 2. 1108 (Olympus, Tokyo, Japan) for time-lapse images. Because cytokinesis often stopped under continuous light, light was manually turned on only during taking images.

### Transmission Electron Microscopy

For TEM, synchronous cultured colonies of *T. socialis* were fixed for 1 h at room temperature with a final concentration of 2% glutaraldehyde obtained by mixing the culture with an equal volume of 4% glutaraldehyde in 0.025 M sodium cacodylate (pH 7.3). Cells were then rinsed with 0.05 M sodium cacodylate buffer for 30 min at room temperature and then added 2% osmium tetroxide in 0.025 M sodium cacodylate buffer (pH 7.3) for 2 h at room temperature. The fixed cells were dehydrated through an ethanol series, replaced by propylene oxide, embedded in Spurr’s resin [46]. Sections were cut with a diamond knife on an Ultracut UCT (Leica, Vienna, Austria) and stained uranyl acetate and lead citrate. These sections were observed with a JEM-1010 electron microscope (JEOL, Tokyo, Japan).

## Supporting Information

Figure S1 **Comparison of distances between basal bodies (BB) and between pro-basal bodies (pBB) in three species of volvocine algae.** (A) Scatter plot of distances between BB (horizontal axis) and between pBB (vertical line). Blue circles, red lozenges and green squares indicate *Chlamydomonas reinhardtii*, *Tetrabaena socialis,* and *Gonium pectorale*, respectively. (B–D) Merged immunofluorescence images of microtubular rootlets and flagella (asterisks) by anti-acetylated tubulin antibody and BB (arrows) and pBB (arrowheads) by anti-CrSAS-6 antibody. Note that two flagella are extended from closer dot pair other than other wider pair. Each scale bar represents 5 µm. (B) *C. reinhardtii*. (C) *T. socialis*. (D) *G. pectorale.*
(TIF)

Figure S2 **Western blot of three species of volvocine algae with antibody against CrSAS-6.** The protein bands were detected with anti-CrSAS-6 antibody in *Chlamydomonas reinhardtii* C-239, *Tetrabaena socialis* NIES-571 and *Gonium pectorale* K4-F3-4. Western blotting analysis was carried out as described by Nakazawa et al. [25] for evaluation of the specificity of the antibody. Western blotting showed that the CrSAS-6 antibody cross-reacted with SAS-6 from the three species. *T. socialis* SAS-6 is slightly larger than those of *C. reinhardtii* and *G. pectorale*.
(TIF)

Video S1 **Swimming of** ***Tetrabaena socialis*** **NIES-571 vegetative colony.**
(AVI)
