# Supplementary figures and images for: The Simplest Integrated Multicellular Organism Unveiled
Source: PLoS One. 2013 Dec 11;8(12):e81641. doi: 10.1371/journal.pone.0081641 (PMC3859500; doi:10.1371/journal.pone.0081641)

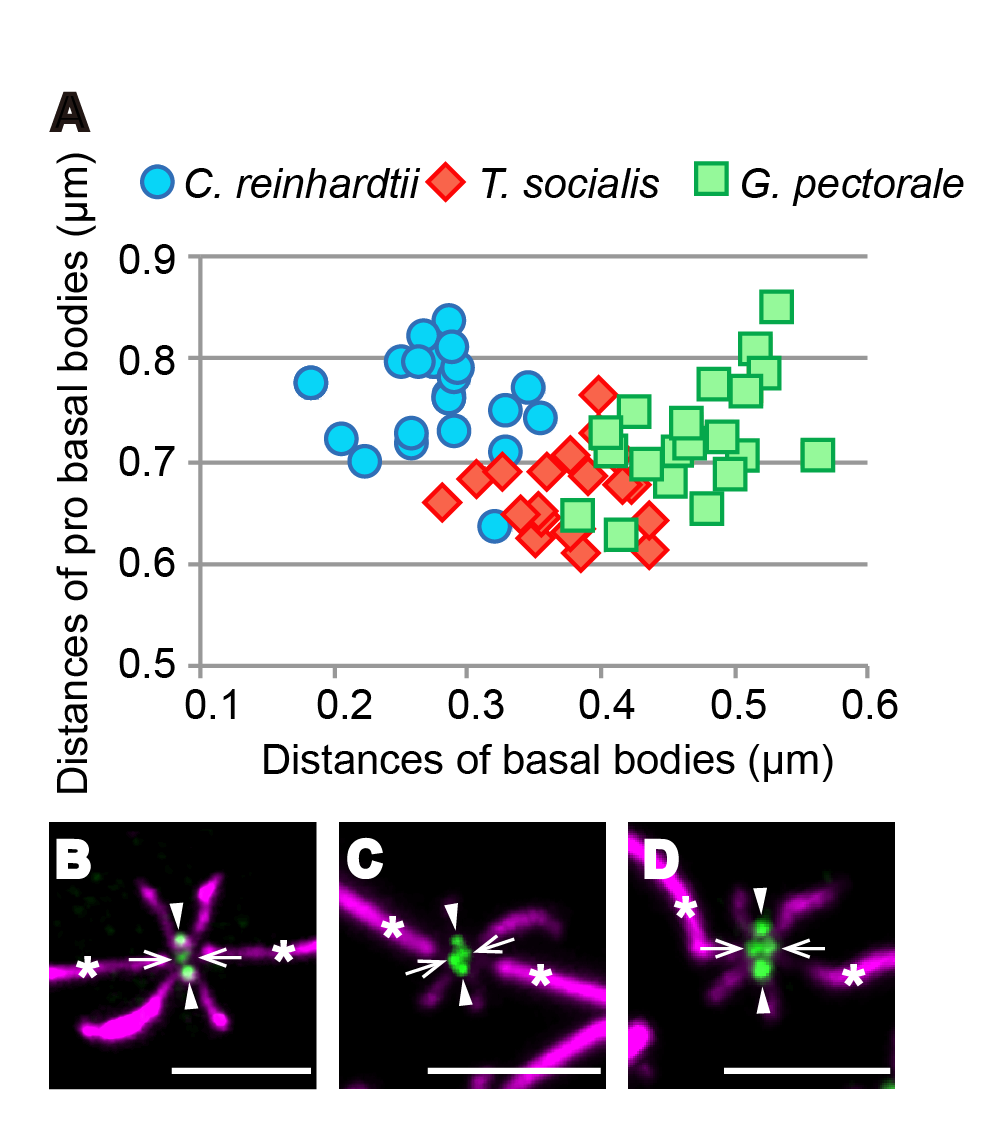

Supplement: Figure S1 — Comparison of distances between basal bodies (BB) and between pro-basal bodies (pBB) in three species of volvocine algae. (A) Scatter plot of distances between BB (horizontal axis) and between pBB (vertical line). Blue circles, red lozenges and green squares indicate Chlamydomonas reinhardtii, Tetrabaena socialis, and Gonium pectorale, respectively. (B–D) Merged immunofluorescence images of microtubular rootlets and flagella (asterisks) by anti-acetylated tubulin antibody and BB (arrows) and pBB (arrowheads) by anti-CrSAS-6 antibody. Note that two flagella are extended from closer dot pair other than other wider pair. Each scale bar represents 5 µm. (B) C. reinhardtii. (C) T. socialis. (D) G. pectorale. (TIF) [file pone.0081641.s001.tif]

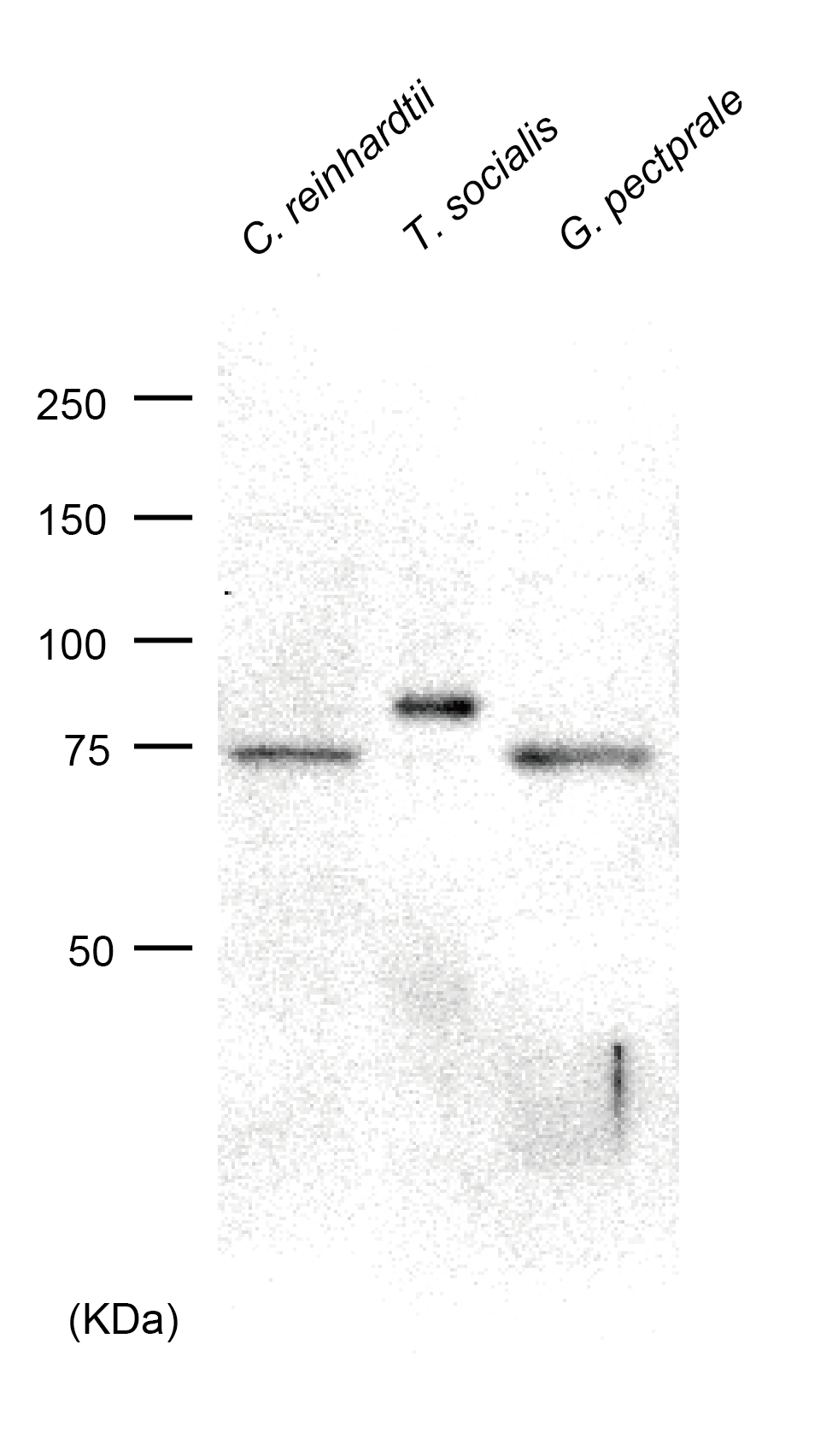

Supplement: Figure S2 — Western blot of three species of volvocine algae with antibody against CrSAS-6. The protein bands were detected with anti-CrSAS-6 antibody in Chlamydomonas reinhardtii C-239, Tetrabaena socialis NIES-571 and Gonium pectorale K4-F3-4. Western blotting analysis was carried out as described by Nakazawa et al. [25] for evaluation of the specificity of the antibody. Western blotting showed that the CrSAS-6 antibody cross-reacted with SAS-6 from the three species. T. socialis SAS-6 is slightly larger than those of C. reinhardtii and G. pectorale. (TIF) [file pone.0081641.s002.tif]
